# Spt4 Promotes Pol I Processivity and Transcription Elongation

**DOI:** 10.3390/genes12030413

**Published:** 2021-03-12

**Authors:** Abigail K. Huffines, Yvonne J. K. Edwards, David A. Schneider

**Affiliations:** Department of Biochemistry and Molecular Genetics, University of Alabama at Birmingham 720 20th Street South, Birmingham, AL 35294, USA; mcconaha@uab.edu (A.K.H.); yedwards@uab.edu (Y.J.K.E.)

**Keywords:** transcription, ribosome, rRNA, RNA polymerase I, Spt4

## Abstract

RNA polymerases (Pols) I, II, and III collectively synthesize most of the RNA in a eukaryotic cell. Transcription by Pols I, II, and III is regulated by hundreds of trans-acting factors. One such protein, Spt4, has been previously identified as a transcription factor that influences both Pols I and II. Spt4 forms a complex with Spt5, described as the Spt4/5 complex (or DSIF in mammalian cells). This complex has been shown previously to directly interact with Pol I and potentially affect transcription elongation. The previous literature identified defects in transcription by Pol I when *SPT4* was deleted, but the necessary tools to characterize the mechanism of this effect were not available at the time. Here, we use a technique called Native Elongating Transcript Sequencing (NET-seq) to probe for the global occupancy of Pol I in wild-type (WT) and *spt4△ Saccharomyces cerevisiae* (yeast) cells at single nucleotide resolution in vivo. Analysis of NET-seq data reveals that Spt4 promotes Pol I processivity and enhances transcription elongation through regions of the ribosomal DNA that are particularly G-rich. These data suggest that Spt4/5 may directly affect transcription elongation by Pol I in vivo.

## 1. Introduction

Transcription is the essential process by which an RNA polymerase (Pol) transcribes a DNA template into RNA. There are three universal eukaryotic Pols: I, II, and III, which primarily synthesize ribosomal RNA (rRNA), messenger RNA (mRNA), and transfer RNA (tRNA), respectively. There are two additional Pols identified in plants, Pol IV, which synthesizes small interfering RNAs (siRNAs) [1], and Pol V, which synthesizes intergenic noncoding (IGN) RNAs [2], and both play a role in gene silencing. Each Pol has a unique cohort of target genes and the regulation of Pols I, II, and III is finely controlled. Whereas Pols II and III have hundreds or thousands of target genes, Pol I transcribes a single gene, the 35S gene in yeast. The transcript produced from the 35S gene by Pol I is co- and post-transcriptionally processed into the three largest rRNAs (18S, 5.8S, and 25S). These rRNAs account for roughly 80% of the total RNA in a rapidly growing cell. In yeast, the ribosomal DNA (rDNA) is organized in approximately 200 tandem repeats on chromosome XII, of which about half are actively transcribed during exponential growth [3]. Transcription factors are universally important for control of transcription by all three Pols [3,4,5,6,7], and many factors have been identified to influence each step in transcription: initiation, elongation, and termination [8,9,10]. These factors play a variety of roles, including recruitment of the polymerase to the promoter region [11,12,13], organization of the DNA template [14,15,16], and binding to termination motifs and promoting release of the polymerase from the template [17,18,19]. Many factors are specific to a single Pol, however, there is a growing list of factors that may play a role in transcription by more than one enzyme [4]. To date, only one transcription factor, TATA-binding protein (TBP), is known to regulate transcription by all three eukaryotic Pols [20].

Spt4 was first discovered nearly 40 years ago [21], and was later identified as a Pol II transcription elongation factor [22,23]. In the earliest studies, it was proposed that Spt4, along with Spt5 and Spt6, could regulate chromatin structure [24]. Later, it was established that Spt4 and Spt5 form the Spt4/5 complex, which can interact with Pol II, and that Spt6 can associate with this complex but is also present in the cell as an individual factor [23]. These findings suggested that the Spt4/5 complex may play a distinct role from Spt6 in transcription by Pol II. Since these early studies, many additional studies have characterized the role of the Spt4/5 complex in transcription by Pol II, and the most widely accepted hypothesis is that this complex may help the polymerase to overcome pausing (especially promoter-proximal pausing), traverse nucleosomes, and aid in nascent RNA processing [25,26,27,28]. Interestingly, Spt4/5 is the only known transcription factor that is conserved throughout all domains of life (with the bacterial homolog of *SPT5* being *nusG*), emphasizing the importance of this factor in transcription in potentially all organisms [29].

In addition to its effects on Pol II, Spt4/5 has been implicated in the control of transcription by Pol I. Previously, both Spt4 and Spt5 were found to copurify with Pol I [30]. Furthermore, in *spt4△* cells, there was a decrease in processivity (the probability that a polymerase will complete transcription without premature termination), a reduction in rDNA copy number, and defects in rRNA processing [30]. In addition to these results, it was discovered that when a point mutation was introduced into *SPT5*, there was a decrease in rRNA synthesis and a severe growth defect in these cells [31]. The *spt5* mutants were viable, but were synthetic lethal with *spt4*∆, suggesting that these two proteins may be able to functionally compensate at least partially for each other. While chromatin immunoprecipitation (chIP) experiments indicated that Pol I processivity was impaired in *spt4△* yeast, electron microscopy (EM) analysis of Miller chromatin spreads did not reveal an obvious Pol I processivity defect in these cells [30]. Therefore, it remains unclear how Spt4 affects transcription by Pol I in vivo.

Here, we utilize a technique called Native Elongating Transcript Sequencing (NET-seq) to interrogate Pol I transcription elongation properties in living cells. This technique was originally developed to investigate the positioning (or occupancy) of Pol II on the DNA template during transcription [32], but was adapted for use with Pol I [33,34]. NET-seq allows for the precise probing of global Pol I occupancy on the rDNA template at single nucleotide resolution in vivo, allowing us to re-examine how Spt4 affects Pol I transcription elongation properties in vivo. We hypothesized that Spt4 would promote transcription elongation (resulting in altered Pol I pausing properties), and that there would be an accumulation of Pol I at the 5′ end of the 35S gene (reflecting impaired Pol I processivity). We found that in *spt4△* yeast, there was a redistribution of reads and an increase in occupancy at the 5′ end of the gene as compared to WT, with this occupancy dropping off at the 3′ end. We further observed repositioning of Pol I throughout the rDNA gene, consistent with an effect of Spt4/5 on Pol I transcription elongation/pausing. These results suggest that, consistent with previous studies, Spt4 is an important transcription elongation factor for Pol I.

## 2. Methods

### 2.1. NET-seq Experiments

Wild-type yeast (WT, described previously [33]) and yeast containing a total deletion of the *SPT4* gene (*spt4△,* described previously [30]) were used for these experiments, with a detailed strain description provided in Table 1. We note that the *spt4△* strain used here is the same strain used in the previous work from the Nomura lab, except an epitope tag was incorporated onto the C-terminus of Rpa135. Deletion of *SPT4* induced a minor change in growth rate (WT doubling time of 100 min versus 110 min for the *spt4△;* Supplementary Figure S2).

Yeast samples were grown, harvested, and lysed as described in previous literature [33]. Immunoprecipitation and RNA extraction were also performed the same, except that after incubating sample with beads for 3 h, beads were resuspended in 900 μL of TES (10mM Tris-HCl pH 7.5, 1 mM EDTA pH 8.5, 1% SDS). Then, two phenol and two chloroform extractions were performed, using 500 μL of either phenol or chloroform. After the final chloroform extraction, 10 μL glycoblue and 40 μL water were mixed, creating the glycoblue solution. Then, 1.4 mL ammonium acetate solution (1M ammonium acetate, 95% ethanol) and 10 μL glycoblue solution were added to each sample, and samples were precipitated for at least 2 h at −80 °C. 

Following precipitation, samples were centrifuged at 4 °C for 1 h at 16,000× *g*. Pellets were washed two times with 750 μL of 75% ethanol. After the last wash, the tubes were left open to allow the pellets to dry completely and for all residual ethanol to evaporate. Pellets were resuspended in 11.5 μL of 10mM Tris-OAc, pH 7.9, with 1 μL used for determining the RNA concentration. Linker ligation and fragmentation were performed as previously published [33], except that a DNA linker with a unique molecular identifier (UMI) was used (5′-/5rApp/CANNNNNNNNCTCCACGAGTCATCCGC/3ddC/-3′, Integrated DNA Technologies). Gel extraction was based on previously published protocol, except that after pulverization, 400 μL of water was added to gel pieces, then tubes were incubated at −80 °C for 30 min and at 70 °C for 20 min. Gel slurries were transferred to Costar Spin-X Centrifuge Tube Filters (Corning) and centrifuged for 3 min at 16,000× *g.* The flow-through was transferred to a new 1.5 mL microcentrifuge tube, and 37.5 μL 3 M ammonium acetate, 1.125 mL 100% isopropanol, and 2 μL glycoblue were added. The samples were precipitated for at least 2 h at −80 °C. 

After precipitation, samples were centrifuged at 4 °C for 1 h at 16,000× *g.* The pellets were washed twice with 750 μL of 75% ethanol. After the final wash, tubes were left open until ethanol was completely evaporated. Once pellets were dry, they were resuspended in 10 μL of 10mM Tris-HCl, pH 6.9. The reverse transcription step was performed exactly as previously published. After reverse transcription, another gel excision was performed as described above, except that slices were cut between 60 and 600 nucleotides. Gel was pulverized, and 500 μL of elution buffer (500 mM ammonium acetate, 1mM EDTA pH 8.0) was added. Gel extraction was performed based on a protocol published by Cold Spring Harbor [35]; gel slurry was incubated at 37 °C for 3 h with nutation. After incubation, slurries were transferred to Costar filtration tubes, centrifuged as before, and flow-through was added to a 1.5 mL microcentrifuge tube with 1.2 mL 100% ethanol and 2 μL glycoblue. Samples were precipitated at −20 °C for at least 2 h.

Samples were centrifuged for 10 min at 4 °C at 16,000× *g.* The ethanol was removed, and tubes were left open until pellets were completely dry and all ethanol was evaporated. Circularization and amplification were performed as previously published [33] with the amplification primers in Table 2, but samples were amplified 25× instead of 12×. Finally, libraries were purified using Aline PCRCleanDX beads, following the manufacturer’s protocol. DNA libraries were sequenced with primers as previously described [33].

### 2.2. Data Analysis

The NET-seq libraries were constructed and sequenced utilizing the Illumina NextSeq500 platform according to manufacturer’s instructions (similarly to the methods in [33]). Following sequencing, the fastq files were preprocessed utilizing three steps. First, the reads were deduplicated based on the UMI sequence using fqtrim (version 0.9.7) with the “-C” option [36] to remove the polymerase chain reaction (PCR) duplicates. Second, to identify the fastq reads on target with the appropriate library format, the reads with the nucleotide sequence (5′-AGNNNNNNNNTG-3′) at the 5′ end were identified. Once these reads were established, the twelve nucleotides were removed, and the remainder of the read was retained. If the read did not begin with this nucleotide sequence, it was discarded. This step was performed using cutadapt (version 1.12; [37]) with the following parameters: “-g AGNNNNNNNNTG –no-indels”. Third, the 3′ library sequence (5′-CTGTAGGCACCAT-3′) was identified and removed using cutadapt [37] with the following parameters “-a CTGTAGGCACCAT –no-indels”. FastQC (version 0.11.4) [38] was used to generate a fastq quality report at the end of each step.

The preprocessed fastq reads were then aligned to the *S. cerevisiae* genome assembly R64-1-1 (GenBank assembly accession: GCA_000146045.2; [39]) using STAR (version 2.7.1a; [40]). The STAR splicing mode was switched off by setting the STAR parameter alignIntronMax to 1. 

SAMtools (version 1.6) was used to sort and index the resulting BAM files [41]. BEDTools (version 2.26.0; [42]) was used to convert the BAM files to BED files and create the genome coverage files from each BED file. The number of 5′ read ends (that correspond to the 3′ end of the originating nascent transcript) mapping to the plus and minus strands for each position in the genome were determined using the BEDTools genomecov function [33]; where the -d -5 parameter was specified to calculate the coverage at the 5′ positions instead of the entire interval. The genome coverage files were generated for the positive and negative strand by configuring the BEDTools genomecov strand parameter to “-strand +” and “-strand −”, respectively [42].

**Figure 1:**
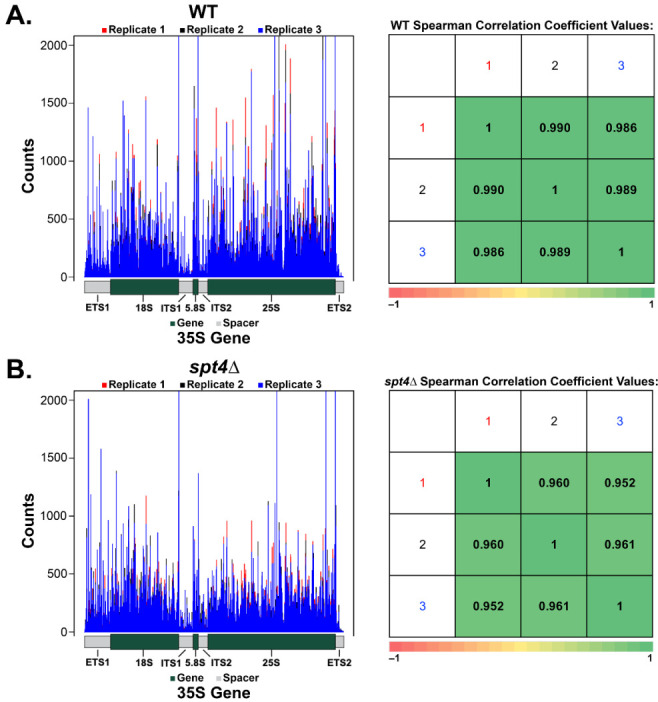
NET-seq experiments are reproducible for WT and *spt4△* yeast strains. Three libraries for each strain were prepared and sequenced. The reads were mapped back to the yeast genome, and were plotted in (**A**,**B**). Spearman correlation coefficient values were calculated comparing each of the three replicates against the other two per strain. A coefficient value of 1 indicates complete similarity between the two replicates (as can be seen when comparing one replicate against itself); whereas a value of −1 indicates completely opposite rank order.

**Figure 2:**
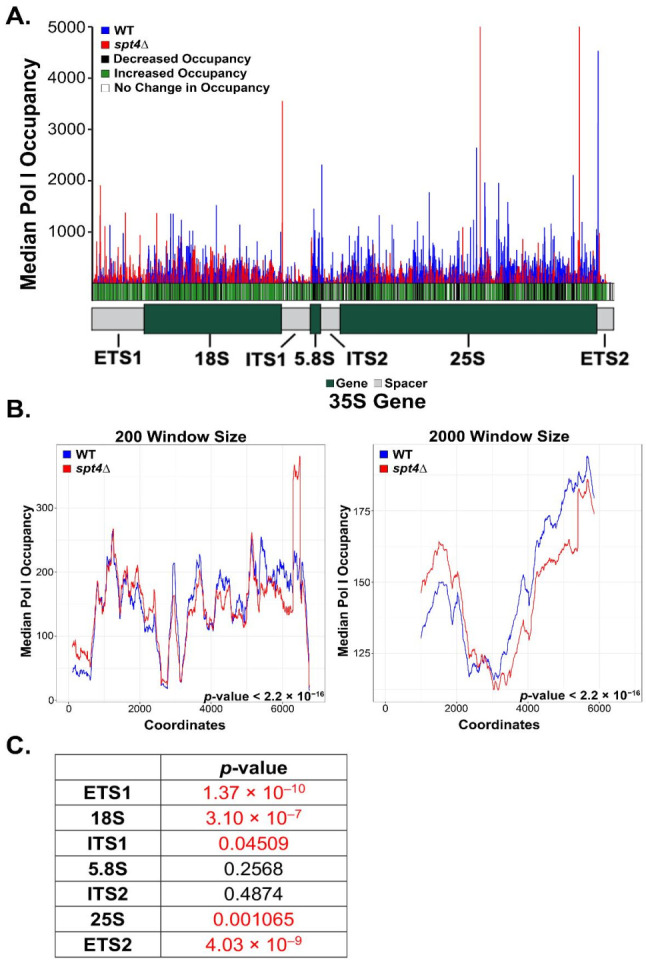
Spt4 promotes Pol I processivity during transcription of the rDNA. (**A**) The median occupancy of the three replicates for each strain was plotted. At each position, a *p*-value was calculated based on the occupancy of the WT vs. *spt4△* strains. If the occupancy in the *spt4△* strain was significantly increased compared to WT, that was deemed “increased occupancy”, and the same process was taken for the “decreased occupancy” positions. (**B**) The moving average was calculated for every x number of positions (where x is equivalent to window size; for the 200 window size, x = 200 and for the 2000 window size, x = 2000), and plotted. The Kolmogorov–Smirnov test was used to determine whether the two strains displayed the same or different distributions; *p*-values are included on the graphs. (**C**) The Kolmogorov–Smirnov statistical test was run for each region of the 35S gene to compare between the occupancy of the two strains. The *p*-value is indicated in the table for each region.

**Figure 3:**
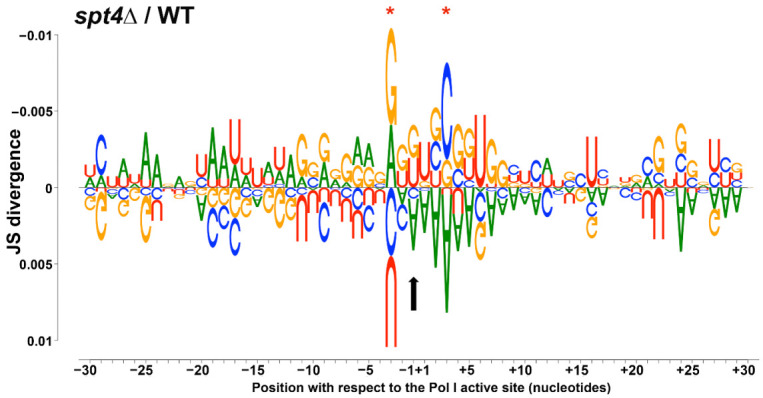
Pol I is stalled in particularly G-rich regions of the rDNA in *spt4△* yeast. A summary logo was generated to demonstrate the differences in sequence-specific pausing in *spt4△* cells versus WT. Sequences 30 nucleotides up- and downstream in the top 2.5% enriched positions were compared using DiffLogo (version 2.14.0). The size of the nucleotide at each position is proportional to the degree of overrepresentation in one strain versus the other. Sequences above the *x*-axis are overrepresented in the mutant strain whereas sequences below the *x*-axis are overrepresented in WT. Positions with statistically significant divergence (see method section) are indicated with a red asterisk, and the position of the last synthesized nucleotide (−1) is marked with a vertical black arrow.

Downstream data processing and visualization of the resulting genome coverage files were carried out using R (version 4.0.2). To generate the histogram plots, sequence logos, and to determine replicate concordance (Figure 1, Figure 2 and Figure 3 and Supplementary Figure S1), the following R packages were used: dplyr (version 1.0.2), plyr (version 1.8.6), ggplot2 (version 3.3.2), ggseqlogo (version 0.1; [43]), ggpubr (version 0.2.5), cowplot (version 1.1.1), matrixStats (version 0.58.0), hexbin (version 1.28.1), tweedie (version 2.3.3), statmod (version 1.4.35), magritter (version 1.5), scales (version 1.1.1), tidyr (version 1.1.2), seqinr (version 3.6-1), zoo (1.8-8). A data frame was created for each sample containing a column for each of the following: chromosome coordinate, coordinate (ascending numbers starting from 1), nucleotide, region (external transcribed spacer 1 (ETS1), 18S, internal transcribed spacer 1 (ITS1), 5.8S, internal transcribed spacer 2 (ITS2), 25S, external transcribed spacer 2 (ETS2)), region type (spacer or gene), sample identity, counts on plus strand, and counts on minus strand. In the yeast genome assembly used, there are two copies of the 35S gene present, so during the generation of this data frame, the count values were combined for the two copies. Reads were normalized by dividing each count value at every coordinate by the total sum of counts on the positive strand. DiffLogo (version 2.14.0; [44]) was used to visualize sequence differences for occupancy of Pol I in WT and *spt4△* yeast cells. For the calculation of a *p*-value for one motif position of one motif pair, DiffLogo computes a *p*-value that two PWM-positions are from the same distribution using permutation tests. Significance indicators in difference logo when *p* < 0.05 (https://rdrr.io/bioc/DiffLogo/src/R/diffSeqLogoSupport.R (accessed on 27 January 2021)). The data used to generate the figures in this publication have been deposited into NCBI’s Gene Expression Omnibus [45], and are available through the GEO series accession number GSE166983.

## 3. Results

### 3.1. NET-Seq Experiments Reproducibly Determine the Occupancy of Pol I on the rDNA Template during Transcription

NET-seq experiments were performed in biological triplicate for both WT and *spt4△* yeast strains. The results from these experiments were plotted and overlaid in Figure 1. The Spearman correlation test was deployed to determine the reproducibility of occupancy patterns between replicates within the same strain. This test ranks occupancy values (i.e., the number of polymerases) at each position in the rDNA template from highest to lowest for two replicates individually, and then compares these rankings. The similarity between the two replicates can be evaluated by the Spearman coefficient value, where a value of 1 indicates perfect similarity. Therefore, the Spearman coefficient value indicates whether the overall occupancy pattern across the 35S gene is similar between replicates. It can be inferred that two replicates resulting in a coefficient value very close to 1 will likely display high and low occupancy in the same regions of the gene. However, because this test takes into account all positions of the 35S gene, conclusions cannot be drawn about individual nucleotides or specific regions of the gene.

In Figure 1A, the three WT replicates displayed a similar occupancy pattern, as indicated by the Spearman correlation coefficient values close to 1. The tall peaks in the graph represent areas of high Pol I occupancy, whereas shorter peaks represent areas that are less occupied by Pol I during transcription. The high peaks may be interpreted as areas where Pol I is moving more slowly (or pausing), whereas areas of low occupancy may indicate where the polymerase is elongating faster, resulting in fewer enzymes being captured at that position. This analytical strategy was repeated for the *spt4△* strain shown in Figure 1B, and the three replicates of this strain were also found to be highly similar compared to each other. Figure 1A,B reveal heterogeneity in occupancy of Pol I across the 35S gene in both strains. These results demonstrate that NET-seq precisely probes for Pol I on the rDNA template during transcription, and that this experiment is reproducible, therefore, giving experimental power to draw comparisons between the occupancy of Pol I in the two strains.

### 3.2. The Occupancy Pattern of Pol I during Transcription Differs in WT and spt4△ Yeast Strains

For a more quantitative comparison, we overlaid the median values for each position of the triplicate datasets for each strain (Figure 2A). At each position, we calculated the *p*-value to determine whether there was a significant difference in occupancy between the two strains, and displayed this result below the histogram [indicated by the green (enhanced occupancy in the mutant); white (no difference in occupancy), and black (reduced occupancy in the mutant)]. We observed that the Pol I occupancy pattern was significantly increased at the 5′ end of the gene and was decreased at the 3′ end of the gene in the *spt4△* strain relative to WT. While NET-seq is a powerful tool for determining transcription elongation occupancy patterns, we cannot determine the absolute number of polymerases that are loaded onto the template (i.e., transcription initiation effects) in the WT vs. *spt4△* yeast from these data. Nevertheless, these data indicate that there is an impairment in polymerase processivity in the *spt4△* strain, revealed by the build-up of polymerases at the 5′ end of the gene.

To visualize trends in occupancy more clearly, we calculated the moving average of median occupancy across the rDNA template for two different window sizes (200 and 2000 base pairs—where the number of positions considered in the average is equal to the window size) for both strains (Figure 2B). These plots support the pattern observed in Figure 2A, where the *spt4△* yeast display an enrichment of Pol I at the 5′ end of the gene and a depletion at the 3′ end compared to WT. We used the Kolmogorov–Smirnov test (K-S test; Figure 2B inset value) to demonstrate that the differences between the mutant and WT patterns are significant. Additionally, we used the same statistical analysis, the K-S test, to compare occupancy distributions in individual rDNA regions between the two strains (Figure 2C). We found that there were significantly different occupancy patterns in the most 5′ end regions (ETS1, 18S, and ITS1) and in the most 3′ end regions (25S and ETS2), while there was no significant difference in the two most interior regions (5.8S and ITS2). Overall, these results suggest that Spt4 promotes Pol I processivity in WT yeast. We hypothesize that in *spt4△* cells, a fraction of the polymerases are prematurely terminating transcription, explaining the significant decrease in Pol I occupying the 3′ end of the gene in the mutant cells. However, it is also possible that slowed elongation kinetics in the 5′ end of the gene contributes to the observed clustering of Pol I in the mutant cells.

### 3.3. Deletion of SPT4 Results in Sequence-Specific Effects on Transcription by Pol I

Figure 1 and Figure 2 suggest that Pol I is pausing or arresting more frequently at the 5′ end of the gene, and could even indicate that some polymerases are lost from the rDNA template. If Spt4 affects transcription elongation properties in addition to processivity, one might detect altered sequence preferences at high occupancy positions in the rDNA. Importantly, NET-seq results display a snapshot of the occupancy of a polymerase at the time of harvest. Thus, kinetic information cannot be directly calculated from these data. However, it is reasonable to interpret peaks and valleys in the NET-seq data as reflections of DNA positions that are slowly transcribed (pause-prone sites) or pause free regions (as outlined above).

To test for sequence-specific effects on Pol I occupancy, sequence logos (Figure S1) for both the WT and *spt4△* yeast strains were generated. For ease of interpretation, we created a logo displaying a summary of the differences in sequence patterns observed between the two strains (Figure 3). Figure 3 and Figure S1 display sequence preferences for the top 2.5% occupied positions in the rDNA, both 30 nucleotides up- and downstream of the last incorporated nucleotide (LNT; black vertical arrow, Figure 3). In Figure 3, nucleotides that are overrepresented in the WT strain are displayed below the *x*-axis, whereas those overrepresented in the *spt4△* yeast with respect to WT are shown above the axis. The degree of difference between the two strains at each position is proportional to the size of the nucleotide.

Interestingly, these data demonstrate that in the *spt4△* strain, the upstream sequence (beyond the RNA:DNA hybrid, roughly beyond −10) was A/T-rich, whereas the downstream sequence contained an enrichment for G/C-content, compared to the WT strain (Figure 3 and Supplementary Figure S1). This downstream G/C enrichment is particularly evident in the first ten positions downstream of the last nucleotide incorporated (+1 to +10; Figure 3). This pattern is the opposite of that observed in the WT strain. We note that only two positions (−2 and +3; Figure 3) gave statistically significant alterations in the sequence motif for occupancy; but given the nature of these data (polymerase position, versus sequence specific DNA binding), it is informative to focus on changes in the observed trends in occupancy rather than specific sequence effects at each position. Ultimately, these data suggest that Pol I occupancy is substantially repositioned with respect to rDNA sequence features. The simplest interpretation of this finding is that Spt4/5 influences transcription elongation kinetics by Pol I, in addition to its effect on processivity.

## 4. Discussion and Conclusions

These studies demonstrate that, consistent with the previous literature, Spt4 is an important transcription elongation factor for Pol I. These results show that in cells lacking Spt4, there was a disruption in Pol I occupancy, where there were relatively more reads at the 5′ end of the rDNA template as compared to WT yeast, and less reads at the 3′ end. Additionally, we saw that in *spt4△* yeast, Pol I populated G-rich regions of the rDNA more densely than WT yeast, where there was an increased A-enrichment downstream of the LNT. The use of NET-seq in these experiments reveals that Spt4 plays a role in transcription elongation by Pol I, and our data suggests that Spt4/5 likely influences both processivity and pausing by Pol I. 

The previous literature suggests that Spt4 promotes Pol I processivity, and it was discovered that in *spt4△* yeast, there was a build-up of polymerases at the 5′ end of the gene and a reduction at the 3′ end, demonstrated by chIP analysis [30]. The data from Figure 2 demonstrate that there was an enrichment of Pol I at the 5′ end of the 35S gene, and that this occupancy dropped off and was significantly lower compared to WT at the 3′ end of the gene. These results are consistent with the previous chIP assays for Pol I processivity. Therefore, the data from our NET-seq experiments support the previous results using alternative techniques [30].

In previous studies using in vitro techniques, it was shown that in *spt4△* mutants, there was impaired transcription elongation by Pol II through G/C-rich regions of DNA [46]. The data presented here are consistent with this previous Pol II study, revealing that Pol I occupancy was enriched in G-rich regions of the rDNA both approximately 10 nucleotides up- and downstream of the LNT (Figure 3). This result may suggest a conserved role for Spt4 in transcription by Pols I and II. In a recent publication, it was determined that in WT cells, peaks of Pol I occupancy correlated with a high G/C-content in the RNA-DNA hybrid present in the transcription bubble [47]. While we do see enrichment of G-residues within this region (−10 to −2) in the WT pause sites (Supplementary Figure S1A), we observe even more G-enrichment in the *spt4△* mutant (Supplementary Figure S1B, and Figure 3). This observation indicates that perhaps, Spt4 functions to help Pol I escape these G/C-rich sequences that may slow the polymerase down. Therefore, in cells lacking Spt4, Pol I is stalled on the template in these regions. That study also showed that the formation of strong nascent RNA structures upstream of Pol I can prevent backtracking and help to propel the polymerase forward. In this study, we determined that the *spt4△* and WT strains display opposing nucleotide occupancy patterns. We predict that the A/T-rich sequences just upstream of Pol I in the *spt4△* cells may form a weak secondary structure. These weak structures could allow for frequent backtracking and stalling of Pol I on the template. 

The exact mechanism by which Spt4/5 promotes transcription by Pol I is not known. Previous studies suggest that the Spt4/5 complex helps Pol II traverse nucleosomes [27,48]. However, unlike the DNA templates for Pol II, the actively transcribed rDNA repeats are thought to be mostly free of ordered nucleosomes [49,50]. Therefore, it is unlikely that the mechanism by which Spt4/5 acts on Pol I and the rDNA is through nucleosome remodeling. Synthesis of rRNA is essential for cell survival, and the previous literature showed that cells containing a point mutation in *SPT5* and a deletion of the *SPT4* gene were unable to survive [31], so it seems that there is some functional redundancy for Spt4 and Spt5. The *spt4△* cells are viable, so we hypothesize that Spt5 can at least partially compensate for the loss of Spt4. 

Another proposed role for the Spt4/5 complex is in reducing pausing throughout elongation. Promotor-proximal pausing is a well-described process and regulation mechanism for Pol II in higher eukaryotes, where the polymerases undergo a strong pause event just downstream of the transcription start site, prior to releasing into transcription elongation [51]. The Spt4/5 complex has been suggested to play a role in this process for Pol II [52]; however, Pol I is not known to undergo this pausing (and there is no evidence for this pausing in our NET-seq data). Therefore, it is unclear whether the Spt4/5 complex could help to resolve polymerase pausing that might occur during elongation. 

Finally, it is possible that the Spt4/5 complex could help to stabilize polymerases on the rDNA. It has been shown previously that Spt4 and Spt5 associate directly with Pol I [30], but it is still unclear whether they interact with the DNA. Some evidence suggests that the Spt4/5 complex may bind to the DNA template in Pol II-transcribed genes [29] while still contacting the polymerase. This is also possible for the rDNA and Pol I, but this structure has not been resolved. It is reasonable to expect that Spt4/5 may influence the structure of the Pol I transcription elongation complex, by enhancing complex stability and processivity. To investigate this further, it would be important to determine where the Spt4/5 complex binds to Pol I through structural analyses and identify its effects on elongation complex structure.

Here, we found that deletion of *SPT4* results in clear perturbation of transcription elongation by Pol I. Consistent with the previous literature [30], these findings support the hypothesis that Spt4 promotes Pol I processivity in vivo, reflected in the accumulation of Pol I at the 5′ end of the 35S gene. Additionally, sequence analyses presented here show that Pol I is most frequently paused in G-rich rDNA regions 10 nucleotides up- and downstream from the LNT, with an elevated A-content upstream of these regions. These data are consistent with previous in vitro studies displaying that Pol II is paused more readily in G/C-rich regions in *spt4△* yeast [46], suggesting that the Spt4/5 complex may have a partially conserved role in transcription by Pols I and II. While these studies reveal more about the role of Spt4 in transcription by Pol I, future studies are required to evaluate the role(s) for Spt5 in Pol I transcription and the connection between Spt4/5 and pre-rRNA processing.

## Figures and Tables

**Table 1:** Description of strains used for NET-seq experiments.

Strain	Description
Wild-Type (WT)	*ade2-1 ura3-1 trp1-1 leu2-3, 112 his3-11,15 can1-100 RPA135-(HA)3- (His)7::TRP1mx6 rpa190Δ::HIS3Mx6* carrying pRS315-*RPA190*
*spt4* *△*	*ade2-1 ura3-1 trp1-1 leu2-3, 112 his3-11,15 can1-100 RPA135-(HA)3-(his)7::URA3mx6 spt4△*::*HIS3*

**Table 2:** Primer sequences for library amplification. The forward and reverse primers for library amplification per replicate are included in the table, and are listed in the 5′ to 3′ direction. The same reverse primer was used for each replicate however, a unique forward primer was used for each.

Rep.	Forward	Reverse
1	CAAGCAGAAGACGGCATACGAGATcagcctcgTCCGACGATCATTGATGGTGCC	AATGATACGGCGACCACCGAGATCTACACtagatcgcCGTCTCTTCTGCGGATGACTCG
2	CAAGCAGAAGACGGCATACGAGATtgcctcttTCCGACGATCATTGATGGTGCC	AATGATACGGCGACCACCGAGATCTACACtagatcgcCGTCTCTTCTGCGGATGACTCG
3	CAAGCAGAAGACGGCATACGAGATtcctctacTCCGACGATCATTGATGGTGCC	AATGATACGGCGACCACCGAGATCTACACtagatcgcCGTCTCTTCTGCGGATGACTCG

## Data Availability

Publicly available datasets were analyzed in this study. This data can be found here: GSE166983 (https://www.ncbi.nlm.nih.gov/geo/query/acc.cgi?acc=GSE166983).

## Supplementary Materials

The following are available online at https://www.mdpi.com/2073-4425/12/3/413/s1, Supplementary Figure S1. Pol I occupies different sequences in WT and *spt4Δ* yeast. Supplementary Figure S2. Growth curves for WT and *spt4Δ* yeast.
