# Supplementary material for: Spt4 Promotes Pol I Processivity and Transcription Elongation
Source: Genes (Basel). 2021 Mar 12;12(3):413. doi: 10.3390/genes12030413 (PMC8000598; doi:10.3390/genes12030413)
Supplement: Supplementary file 1 [file genes-12-00413-s001.pdf]

SUPPLEMENTARY FIGURES:

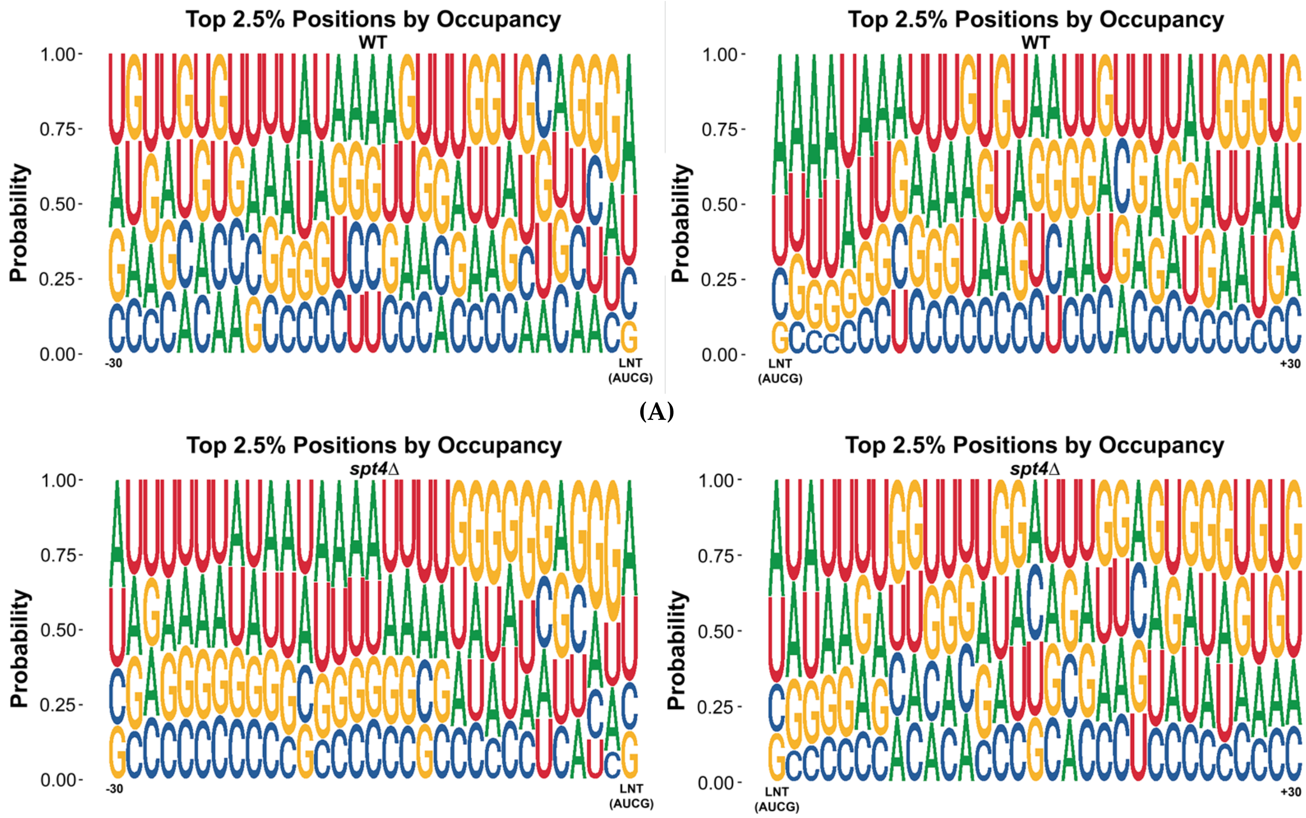

(B)

Supplementary Figure S1. Pol I occupies different sequences in WT and spt4⊿ yeast. Sequence logos were generated for both (**A**) WT yeast and (**B**) spt4⊿ yeast for 30 positions upstream of the LNT (left) and for 30 positions downstream (right).

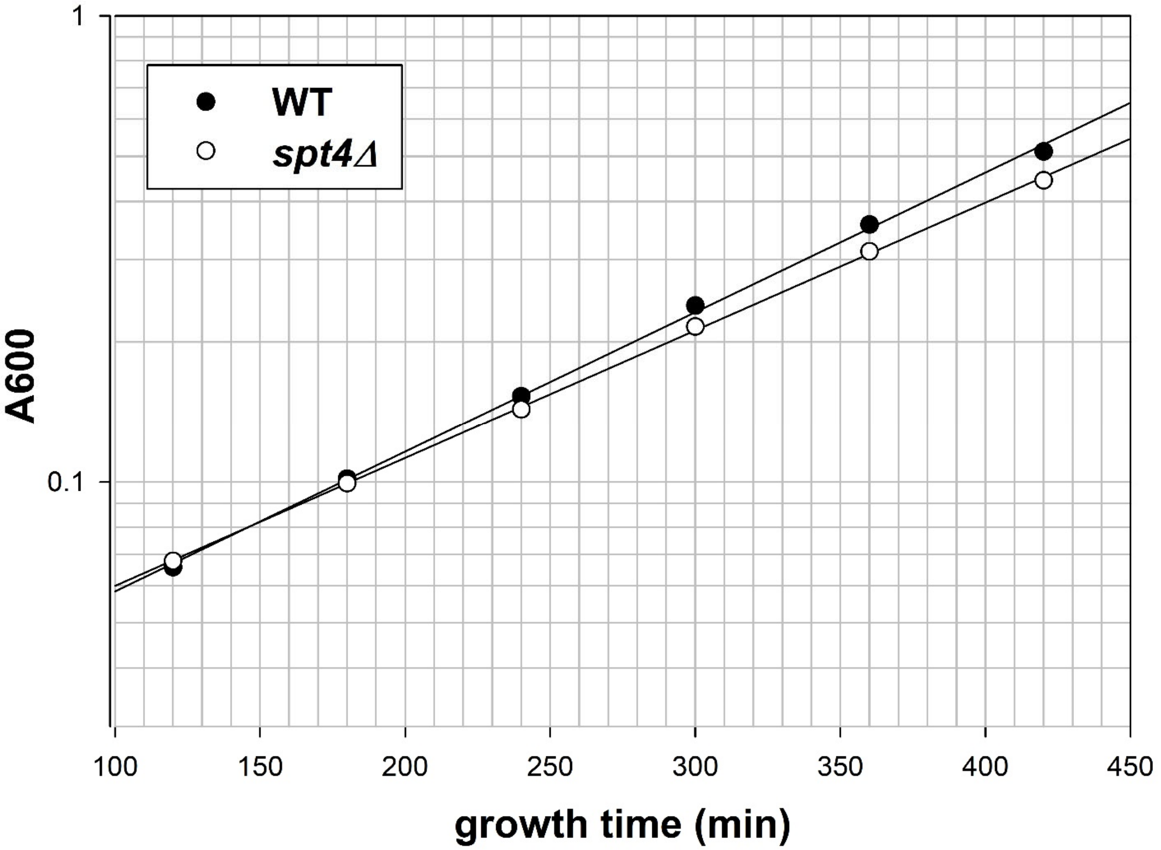

Supplementary Figure S2. Growth curves for WT and *spt4Δ* yeast. Four independent cultures of each strain were cultures at 30 °C with aeration in YEPD medium and cell density was assess my measuring absorbance at 600 nm by spectrophotometry. The resultant calculated doubling times were 100 min for WT and 110 min for *spt4Δ*.
